# Chemical Composition, Total Phenols and Flavonoids Contents and Antioxidant Activity as Nutritive Potential of Roasted Hazelnut Skins (*Corylus avellana* L.)

**DOI:** 10.3390/foods9040430

**Published:** 2020-04-04

**Authors:** Stefan Ivanović, Nataša Avramović, Biljana Dojčinović, Snežana Trifunović, Miroslav Novaković, Vele Tešević, Boris Mandić

**Affiliations:** 1 National Institute, Institute of Chemistry, Technology and Metallurgy, University of Belgrade, Njegoševa 12, 11000 Belgrade, Serbia; stefan.ivanovic@ihtm.bg.ac.rs (S.I.); bmatic@chem.bg.ac.rs (B.D.); mironov@chem.bg.ac.rs (M.N.); 2 Faculty of Medicine, Institute of Medical Chemistry, University of Belgrade, Višegradska 26, 11000 Belgrade, Serbia; 3 University of Belgrade—Faculty of Chemistry, Studentski trg 12-16, 11000 Belgrade, Serbia; snezanat@chem.bg.ac.rs (S.T.); vtesevic@chem.bg.ac.rs (V.T.); borism@chem.bg.ac.rs (B.M.)

**Keywords:** hazelnut skins, phenols and flavonoids content, DPPH-scavenging effect, antioxidant activity, lipids, proteins, carbohydrates, metals and C, H, N, S elements

## Abstract

The present study evaluates natural composition of Serbian roasted hazelnut skins (HS) with potential role in application as functional nutrient of various food products. Total phenols (TPC) and flavonoids contents (TFC) in HS extracts obtained with different ethanol concentrations (10%—I, 50%—II and 96%—III) and their antioxidant activities were investigated. The highest total phenols content (706.0 ± 9.7 mgGAE/g_extract_) was observed in 96% ethanol HS extract. Ethanol HS extracts showed very high antioxidant activity with effective concentrations (EC_50_) ranged between 0.052 and 0.066 mg/mL. The phenol and flavonoid content of roasted HS extracts I–III was determined by HPLC-ESI-MS/MS analyses. Contents of lipids, proteins, carbohydrates, metals, and C, H, N, S elements in roasted HS were also determined. Relatively high C/N, C/P and C/N/P ratios, rich metal contents and fatty acids composition indicated that hazelnut skin might be a good candidate for use as either human or fungal functional nutrient. In addition, possible application of phenolic HS extracts as UV booster was studied by recording UV spectra (220–440 nm) of 10 mg/L of HS extracts I–III combined with 10 mg/L of chemical sunscreen agent benzophenone-3 and *in vitro* sun protection factor (SPF) was calculated.

## 1. Introduction

Hazelnuts are one significant commercial crop in the past decade due to the great world application in confectionery industry (dairy, bakery, and chocolate products), as well as in cosmetics and pharmaceuticals. Leading global producer of hazelnuts is Turkey, followed by European Union countries and the USA [1,2].

Serbia cultivates hazelnuts on approximately 2.200 ha and produces about 800–1000 t, which is actually not enough to meet local demands [3]. There is a growing interest in domestic hazelnut production because of favorable agricultural conditions, but it should be supported by the adequate choice of cultivars with long period of investments and incoming yield from fifth until eighth years of cultivation [4]. Hazelnut cultivars in Serbia come from the European hazelnut (*Corylus avellana* L.) and are mostly grown in Vojvodina, in the north of Serbia.

It is well known that 90% of hazelnuts (*C.avellana* L.) are used in food industries as shelled nuts [5,6], and during hazelnut industry production some by-products are produced (shell, green leafy cover, and skin). Actually, hazelnut skins are the main by-product after roasting in the hazelnut industry which account for 2.5% of the total nut weight [7,8]. Some studies have reported that this waste material can find application as functional food ingredients regarding hazelnut skin richness in polyphenols and dietary fiber [7,9]. Recently published data indicated that roasted hazelnut skin contained the highest content of total phenolics and has the highest antioxidant activity, followed by natural and roasted hazelnuts [8]. Lately, the antioxidant effect of hazelnut skin attracts great attention showing that by addition to coffee, bread, and yogurt improved their physiologically positive effect and antioxidative activity [10,11,12]. The hazelnut skin extracts also revealed functional activity as ingredients in bacterial media for two probiotic strains (*Lactobacillus plantarum* P17630 and *Lactobacillus crispatus* P17631), improving significantly their growth [13]. Alfonso et al. reported that antioxidants such as vitamin C, vitamin E, and coenzyme Q10 might decrease skin damage caused by absorption of UV-radiation, by enhancement of photo-stability and *in vitro* sun protection factor (SPF) of chemical sunscreen agent avobenzone [14]. Devehat et al. also revealed synergistic effect of antioxidant such as lichen extract with sunscreen agent azobenzone that intensify its SPF [15]. Galanakis et al. showed potential of application of recovered phenols from olive mill wastewater as UV booster in cosmetics and possibility to reach desirable SPF value by addition of estimated amount of olive phenols [16,17]. 

To the best of our knowledge, there is no literature data about natural composition of Serbian hazelnut skins. Therefore, the object of our research was to study the natural composition of domestic roasted hazelnut skin, extracts, obtained with various ethanol concentrations and their antioxidant activity. In order to evaluate the possibility of application of this industrial by-product as functional food ingredients total content of phenolics, flavonoids, fatty acids, proteins, carbohydrates and minerals was examined. Identification and determination of phenol compounds in HS extracts was performed by HPLC-ESI-MS/MS analyses with usage of phenolic and flavonoid mixture of standards. Results obtained for Serbian HS extracts are compared with literature data for HS from different geographical origin. Based on the fact that phenols of roasted HS absorb in UV region, the possible potential of using phenolic extracts recovered from HS as UV protective booster in cosmetics was studies as well.

## 2. Materials and Methods 

### 2.1. Chemicals

Folin–Ciocalteu reagent, catechin monohydrate, gallic acid monohydrate, ascorbic acid (vitamin C), 1,1-diphenyl-2-picrylhydrazyl (DPPH), and 2,6-di-tert-butyl-4-methylphenol (BHT) used for determination of total phenol content and antiradical activity were purchased from Sigma-Aldrich. Standards of 20 phenolic compounds: galic acid, protokatehuic acid, *p*-hydroxybenzoic acid, catechin, vanilic acid, chlorogenic acid, caffeic acid, epicatechin, syringic acid, vanillin, *p*-coumaric acid, ferulic acid, sinapic acid, rutin, resveratrol, ellagic acid, quercetin, naringenin, myricetin, and kaempferol, were purchased from Sigma-Aldrich (Steinheim, Germany), Fluka Chemie GmbH (Buchs, Switzerland) or from Chroma Dex (Santa Ana, CA, USA). All chemicals and solvents (n-hexane, chloroform, ethanol, methanol, formic acid) were of reagent-grade level and purchased either from Sigma-Aldrich.

### 2.2. Hazelnuts Skin (HS) Sample

Hazelnuts skin sample (production waste material) was obtained from Tira DOO company, which suppliers with raw hazelnuts (Tonda Gentile Romana variety, *Corylus avellana* L.) are local producers from Prhovo and Pecinci (villages near Stara Pazova, Serbia). The roasted process was conducted at120 °C for 20 min in an industrial continuous-working oven, where the skins were separated from the roasted kernels by vigorously rubbing them against themselves, followed by skin removal via vacuum. Hazelnut skins (HS) were milled and sieved to obtain a 35 mesh (0.5 mm) sieve using an ultra-centrifugal mill. The representative powdered hazelnut skin sample was obtained by the coupe and the chess field method.

### 2.3. Proximate Composition of HS Sample

Moisture, ash, as well as protein, lipid and carbohydrate contents in HS powered sample were determined. The moisture and ash contents were determined using standard methods [18]. Total nitrogen and total protein contents (conversion factor: 6.25) were obtained using Kjedahl digestion (BUCHI Auto Kjeldahl Unit K370 and BUCHI Speed Digester K439). Lipid fraction was extracted using a semiautomatic Soxhlet Buchi Extraction System B-811 (Buchi Labortechnic AG, Flawil, Switzerland) with petrolether as the extraction solvent. Carbohydrates content was calculated by subtracting contents of other compositions from 100%. Analyses were performed in triplicate and the data were calculated as a percentage of the dry weight of the HS powder.

### 2.4. Elemental Analysis of HS 

The carbon, nitrogen, hydrogen and sulfur contents of HS were estimated by elemental analysis using a 2003 Vario EL III Elemental analyzer (CHNS-O; Elementar Analysensysteme GmbH, Hanau, Germany). CHNS analysis was performed as follows: each separate sample (HS in triplicate) dropped into the catalytic tube of the instrument and underwent combustion at 1120 °C in an oxygenated atmosphere. Helium was used to carry the resulting gases (CO_2_, N_2_, H_2_O and SO_2_), through specific adsorption columns where they were separated from each other to a thermal conductivity detector. WinVar software was used to calculate a percentage of each element from concentration of corresponding gases. The daily factors of elements were determined using sulfanilic acid (Merck, standard for elemental analysis) and they were used for results correction.

### 2.5. Metals Contents of HS 

The digestion of HS was performed on Advanced Microwave Digestion System (ETHOS 1, Milestone, Sorisole (BG), Italy) using HPR-1000/10S high pressure segmented rotor. For total mineralization, about 0.6 g of HS powder sample was precisely weighed with accuracy ±0.1 mg and mixed with of 10 mLHNO_3_ (70 wt. %, ACS reagent, Sigma Aldrich, Darmstadt, Germany) and 1 mLH_2_O_2_ (30 wt. %, ACS reagent, Sigma Aldrich, Darmstadt, Germany). The suspension was then heated with microwave energy for 30 min. The temperature was gradually raised to 200 °C in the first 10 min, remained at 200 °C in the next 20 min, and then decreased rapidly to room temperature. After cooling and without filtration, the solution was diluted to a fixed volume into volumetric flask of 25 mL with ultrapure water. Ultrapure water with a resistivity of 18.2 MΩ·cm (equal to 0.05 µS/cm) was prepared using a Barnstead™ GenPure™ Pro (Thermo Scientific, Germany). The contents of major and trace (essential and nonessential) elements were determined by inductively coupled plasma optical emission spectrometry, ICP-OES (Thermo Scientific iCAP 6500 Duo ICP, Thermo Fisher Scientific, Cambridge, United Kingdom). The calibration solutions were made from certified plasma standard solutions: Multi-Element Plasma Standard Solution 4, Specpure^®^, 1000 µg/mL and Mercury, plasma standard solution, Specpure^®^, Hg 1000 µg/mL (Alfa Aesar GmbH & Co KG, Karlsruhe, Germany) and ILM 05.2 ICS Stock 1 (VHG Labs, Inc.- Part of LGC Standards, Manchester, NH 03103 USA). For each digested sample, the ICP-OES measurement was carried out in triplicate (*n* = 3). The reliability of measurements was approved by relative standard deviation lower than 0.5%.

### 2.6. Total Lipid Extraction 

Total Lipid extracts (TL) were obtained by extraction of 5 g of HS powder with 100 mL of hexane and chloroform/methanol (2:1, *v*/*v*). After 30 min of extraction on ultrasonic bath (Sonorex Super RK 100, Bandelin, Berlin, Germany), extracts were left in darkness for 24 h. The extracts were filtrated and HS residues were re-extracted with 20 mL of above-mentioned solvents. Extracts were combined and dried in a rotavapor. The extraction yield was expressed as dry matter percentage.

### 2.7. Extraction of Phenolic Fraction

HS samples were defatted with n-hexane and after that, the extraction of phenolic compounds was performed using different concentrations of ethanol: 10% (I), 50% (II) and 96% (III). Solid/liquid ration was 1/10 (*w*/*w*). After 15 min of extraction on ultrasonic bath, they have left at room temperature over the night. Then, solutions were filtrated through a filter paper and extracts were washed with 20 mL of corresponding solvents, with which they were previously extracted. Extracts were collected and dried in a rotavapor. The extraction yield was expressed as dry matter percentage.

### 2.8. NMR Spectra of TL and Phenolic Extracts 

^1^H NMR spectra of hexane, chloroform/methanol (2:1, *v*/*v*) TL extracts and phenolic fractions I–III were recorded by a Bruker AVANCE III 500 spectrometer (500.26 MHz for ^1^H) equipped with 5mm broadband probehead (BBO). The spectra were measured at room temperature (298K) in CDCl_3_for all hexane and chloroform/methanol TL extracts, while spectra of phenolic fractions I–III were measured in CD_3_OD/D_2_O.

### 2.9. Total Phenols Content 

Total phenol contents (TPC) in the roasted HS sample were determined by the Folin–Ciocalteu method based on already described procedure [19] with some modification [20]. Briefly, 50% ethanol solutions of HS extracts (I–III) were prepared with following concentrations: 0.025, 0.05 and 0.1 mg/mL, as well as, a Folin–Ciocalteu’s reagent by dilution with water (1:9). 0.25 mL of HS samples was mixed with 1.25 mL of Folin–Ciocalteu’s reagent and 1 mL 7.5% of sodium carbonate solution. The reaction mixtures were kept in the dark for 60 min. Absorbance of reaction mixtures was read at 725 nm using GBC Cintra 40 UV–VIS spectrometer (GBC Scientific Equipment, 2002). A blank sample contains distilled water instead of HS ethanolic extracts. Total phenol contents of each HS ethanolic extract (I–III) were determined from standard curve constructed with gallic acid as a standard in the concentrations of: 0.1, 0.075, 0.05, 0.025, and 0.01 mg/mL.

### 2.10. Total Flavonoid Content

Total flavonoid content (TFC) was determined by the method described by Kemtekar et al. [21], with some modifications. HS ethanolic extracts I–III were prepared with following concentrations: 0.075, 0.1, and 0.2 mg/mL. Volume of 300 µL of appropriate HS extract were mixed with 1200 µL of distilled water and 90 µL of 5% NaNO_2_ and after 5 min, 90 µL of 10% AlCl_3_ were added. Then after 6 min 600 µL of 1M NaOH and 720 µL of distilled water were added, and the mixture was kept in the dark for 25 min. Absorbance of yellowish color supernatant was measured at 510 nm by UV/VIS spectrophotometer using methanol as control. A calibration curve was performed with different concentrations of catechin (0.05, 0.1, 0.2, 0.3, and 0.4 mg/mL) dissolved in methanol. The results were expressed as mg catechin equivalent (CE) per g of extract.

### 2.11. Antioxidant Capacity 

#### 2.11.1. DPPH Radical-Scavenging Capacity of n-Hexane and Chloroform/Methanol TL Extracts

The capacity to scavenge the DPPH free radical was monitored according to the method described by [19], and slightly modified [22]. Several concentrations of n-hexane and chloroform/methanol HS extracts diluted with toluene (0.4 mL) were mixed with 1.6 mL of toluene solution containing DPPH radicals (1 × 10^−4^ mol/L). The reaction mixtures were kept in the dark for 30 min at room temperature and absorbance of reaction mixtures was read at 515 nm using UV/VIS spectrometer. A blank sample contained toluene instead of HS extracts. DPPH-scavenge effect of each HS ethanolic extract (I–III) was determined from standard curve using 2,6-di-tert-butyl-4-methylphenol (BHT) toluene solution as a standard, with following concentrations: 0.25, 0.50, 0.75, 1.00, 1.25 and 1.50 mg/mL. Results were expressed as equivalents of BHT providing 50% inhibition (EC_50_) which were calculated from the graph of scavenging effect percentages (absorbance) against concentrations of standard.

#### 2.11.2. DPPH Radical-Scavenging Capacity of HS Ethanolic Extracts (I–III)

The capacity to scavenge the DPPH free radical was monitored similar to the method described above in Section 2.11.1 [20]. Several concentrations of HS ethanolic extracts I–III were prepared with following concentrations: 0.0125, 0.05, 0.075, 0.1 and 0.2 mg/mL. 0.2 mL of each HS methanol sample was mixed with 1.8 mL of methanol solution containing DPPH radicals (0.04 mg/mL). The reaction mixtures were kept in the dark for 30 min at room temperature and absorbance of reaction mixtures was read at 517 nm by UV/VIS spectrometer. A blank sample contained methanol instead of HS extracts. DPPH-scavenge effect of each HS extract was determined from standard curve using vitamin C as a standard. Results were expressed as equivalents of vitamin C providing 50% inhibition (EC_50_), which was calculated from the graph of scavenging effect percentages (absorbance) against concentrations of standard.

### 2.12. HPLC-ESI-MS/MS Analysis

Phenolic compounds identification and determination in HS extracts I–III were performed by Agilent HPLC 1200 Series (Agilent Technologies, Palo Alto, CA, USA) comprising of a vacuum degasser (G1379B series), binary pump (G1312B), autosampler (G13670) and thermostatted column compartment (G1316B), coupled to an Agilent Technologies 6410B Triple Quad LC/MS.HS extracts **I–III** were dissolved in methanol to a concentration of 20 mg/mL, filtered through a 0.45 µm syringe filter and prepared for recording by ten times dilution.

Data acquisition and processing were developed by Agilent Mass Hunter B.06.00 software, using Dynamic MRM software mode with a retention time window of 2 min. The injection volume was 2 μL, and the chromatographic separation was carried out with an Agilent Zorbax Eclipse XDBC18 column, 4.6 mm × 50 mm × 1.8 μm at a temperature of 40 °C. The system employed 0.1% formic acid in methanol (mobile phase A), and 0.1% formic acid in milli Q water (mobile phase B) with the following gradient: 95% of B for 2 min, a linear gradient up to 50% of B in 10 min, to 30% of B in 12 min, to 10% of B in 20 min and finally an isocratic mode at 10% of B for 5 min. Finally, an equilibration step coming back to 95% of B during 5 min was performed. The system was provided with an electrospray ion source, employing nitrogen as the nebulizer gas. This ion source was configured as follows: 325 °C for the gas temperature, 10 L/min for the gas flow rate, 40 psi for the nebulizer pressure, 4000V for the capillary voltage in negative mode. The MS used nitrogen as the collision gas (99.999% purity). The calibration curve was prepared with mixture of 20 standards solution, ranging from 0.02 to 5 µg/mL. 

### 2.13. Total Fatty Acid Analysis

Tridecanoic acid was added to TL extracts as internal standard. After methylation with 1M sulfuric acid in methanol for one hour at 85 °C [23], samples were analyzed by gas chromatograph (Shimadzu 2014, Kyoto, Japan) on Rtx 2330 columns (Restek, Bellefonte, PA, USA). Initial column temperature was set at 140 °C and hold for 5 min. Then temperature was increased to 220 °C by 3 °C/minute. Final column temperature was held for 20 min. Peaks of fatty acids (FA) in samples were identified by comparing the retention times with those obtained for FA standard mixtures PUFA-2 and Supelco37 Component FAME Mix (Sigma-Aldrich, Darmstadt, Germany). Quantity of identified FA in lipid extract was calculated by comparing their areas in chromatographs with internal standard peak area in sample. 

### 2.14. In Vitro Sun Protection Factor (SPF)

The UV spectra in the range 220–400 nm wavelength of chemical sunscreen agent benzophenone-3, HS ethanolic extractsI–III and combined benzophenone-3 and HS extracts I–III, dissolved at concentration of 10 mg/L in 96% ethanol, were recorded by using GBC Cintra 40 UV–VIS spectrometer (GBC Scientific Equipment, 2002). SPF factors were determined by modified method of Galanakis et al. [16,17] applying their spectra in UVB range between wavelength of 290 and 320 nm. The following equation was used to calculatein vitroSPF:

$$SPF\;spectrophotometric=CF\times \sum_{290}^{320}EE(\lambda )\times I(\lambda )\times Abs(\lambda )$$

where *EE*(*λ*) is the erythemal effect spectrum, *I*(*λ*) is the solar intensity spectrum, *Abs*(*λ*) is the absorbance of the sunscreen, *CF* is correction factor equal to 10. The constants of *EE*(*λ*) × *I*(*λ*) were obtained by Sayre et al. [24].

### 2.15. Statistical Analysis

Results were expressed as means ± standard deviation (*n* = 3) for each determination. The statistical significance among different extracts was estimated by one-way analysis of variance (ANOVA) followed by comparing results with calculated LSD (least significant difference) values. Student *t*-test was used to determinate the significance of the differences between the mean values. The statistical analysis was performed using Microsoft Excel statistical software (Microsoft Excel 2007). Differences at *p* < 0.05 were considered to be significant. 

## 3. Results and Discussion 

### 3.1. Proximate Composition of HS Sample

The proximate composition of roasted HS sample is given in Table 1. Moisture, proteins and ashes contents of HS were shown on dry weight (dw) basis and they are similar to reported data [11,25,26]. The lipid content is in the range of 11.0–18.8% from Georgia (11%) to Tombul (14.5%), Tonda Gentile Trilobata (17.2%) and San Giovanni (18.8%) cultivars of HS samples [12,25]. Carbohydrates are the major components of HS which are in close agreement with the study of Bertolino et al. [12]. 

### 3.2. Elemental Analysis and Metals Contents of HS

Elemental analysis of HS sample was performed showing contents of 54.280 ± 0.185%C, 6.005 ± 0.097%H, 1.278 ± 0.019%N and 0.268 ± 0.048%S. 23 minerals (essential and nonessential) of HS sample were determined by ICP-OES and summarized in Table 2. Magnesium and calcium are the most abundant minerals, followed by sodium, phosphorous and potassium. HS sample is also good source of chromium, iron, manganese, nickel, and selenium, while toxic metals arsenic, lead and mercury are not detected (Table 2). Presence of significant amounts of essential minerals confirmed health status of HS and its nutrient value. Content of trace mineral selenium of 23.9 µg/100 g is especially important because it has crucial antioxidant role in protection of cell membranes by annulling deleterious effects of free radicals (ROS) [27]. The highest content of selenium was reported for Sicilian hazelnut (86.5 µg/100 g) and Turkish Tombul hazelnuts (60 µg/100 g) [28,29]. The C, N, and P contents of HS are in the range of fungal biomass which varied from 38 to 57%, 0.23 to 15%, and 0.040 to 5.5%, respectively [30], indicating that HS might be a good nutrient for fungal growth. Fungi are known to have relatively higher C/P and C/N ratios, compared to bacteria, which may be related to their large size and small surface area to volume ratio, which decreases the relative demand for P-rich membrane phospholipids [31,32]. Therefore, high C/P ratio (1044) and C/N ratio (42.5) of HS also demonstrated its potential application as nutrient for fungal growth.

### 3.3. TL and Phenolic Extraction

TL extracts obtained by extraction with hexane and chloroform/methanol (2:1, *v*/*v*) gave yields of 13.5% and 15.6% of dry weight, respectively. Higher yield of chloroform/methanol TL extract derived from the presence of polyphenols due to the higher polarity of solvent. Extraction of phenolic compounds is significantly dependent on solvent, degree of phenolic compounds polymerization, as well as the effect of lipid interfering compounds on phenols [20]. Many different solvents such as aqueous methanol, ethanol, acidified methanol, acidified ethanol, and acetone were used for phenolic extraction [6,26,33,34,35] and aqueous acetone solution was shown as the best solvent because it extracted phenolics in a higher yield that ethanol and methanol [6,26]. In our studies, different concentrations of water solutions of ethanol were used for phenolic extraction: 10% (I), 50% (II), and 96% (III). Phenolic extraction yield, expressed as grams per 100 g of defatted samples, was in the order: III (27.2%) > II (18.5%) > I (15.7%) and it increases with increasing concentration of ethanol. Actually, it was reported for all plant materials that the highest yields of phenolic compounds were observed by using ethanol/water 50/50 (*v*/*v*) and acetone/water 50/50 (*v*/*v*) solutions as solvents [36]. Phenolic extraction yields I–III are similar with reported data (27.95%) for using ethanol as an extraction solvent, while they have lower values compared to results for other extraction solvents (27.95–43.67%) [26].

### 3.4. NMR Data of TL and Phenolic Extracts 

^1^H NMR spectra of hexane and chloroform/methanol TL extracts confirmed the presence mostly triglycerides with characteristic chemical shifts of vinylic, mono-allylic, bis-allylic and alkyl hydrogens corresponding to the fatty acids (oleic, linoleic, linolenic, palmitic and stearic acids) as in reported data [37]. The spectra of phenolic extracts I–III revealed the presence of polyphenols beside triglycerides regarding to appearance of chemical shifts of aromatic ring protons in the range 5.6–9.0 ppm [38].

### 3.5. Total Phenols and Flavonoids Contents of HS

**Figure 1:**
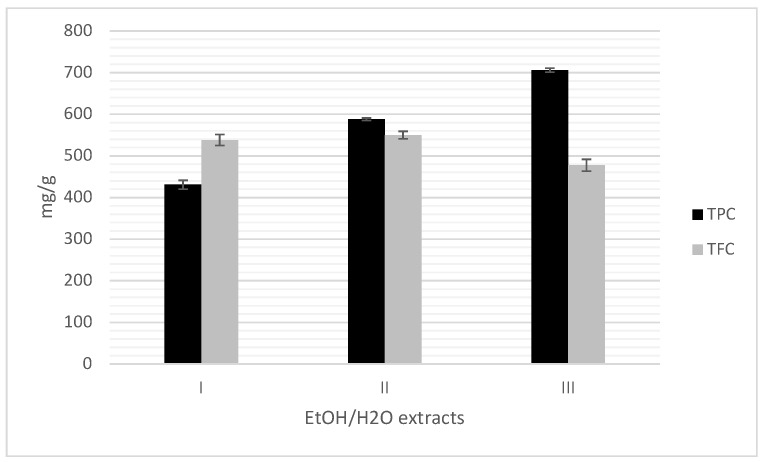
Total phenols (mg GAE/g) and flavonoids (mg CE/g) contents in HS extracts I–III.

Total phenols contents (TPC), expressed as mg of GAE per gram a dry sample, are shown in Table 3 and Figure 1. The content of phenolic compounds (GAE/g) was in the following order: I < II < III (Figure 1), indicating that with increasing concentration of ethanol, the content of total phenols had a higher value. The TPC values are similar with reported results of 638 and 706 mg GAE/g for medium roasted and high roasted HS samples, respectively, by using the same solvent ethanol [26]. The TPC have lower values for fourteen Turkish varieties ranged between 51.9 and 203.1 mg GAE/g [7], while for HS using aqueous methanol (80/20, *v*/*v*), aqueous ethanol (80/20 *v*/*v*) and aqueous acetone (80/20, *v*/*v*) for extraction, the obtained values are 499.7, 588.2 and 546.6 mg CE/g, respectively [39]. Total flavonoid contents (TFC) of HS extracts I–III, expressed as mg of CE per gram sample are very similar as it is shown in Table 3 and Figure 1. The highest yield of total flavonoids was observed in 50% ethanol HS extract II. Compared to TFC of HS of fourteen Turkish varieties which are in the range of 30.9–112.4 mg CE/g, TFC of HS (I–III) have approximately 5 to 17 times higher values [7] which might be caused by different solvents and extraction methods as well as different varieties, geographic origin and harvest year of the samples [26].

### 3.6. Antioxidant Activity

Antioxidant activity of hexane and chloroform/methanol TL extracts, as well as, HS extracts I–III was determined by DPPH assay. The scavenging effect of HS extracts I–III was expressed as EC_50_ values, as mg sample/mL required to scavenge 50% of DPPH radicals and as equivalents of vitamin C, shown in Table 3. The lower EC_50_ value means the HS sample has higher antioxidant activity. 

The synthetic antioxidant BHT (butylated hydroxytoluene) and natural antioxidant vitamin C (ascorbic acid) were used as reference for TL extracts and HS extracts I–III. Hexane and chloroform/methanol TL extracts showed antioxidative activity of EC_50_ = 11.09 mg/mL and EC_50_ = 3.35 mg/mL, respectively. The BHT had EC_50_ of 1.88 mg/mL under the same conditions. Therefore, hexane and chloroform/methanol TL extracts exhibit DPPH scavenging activity that is almost 6 and 2 times weaker than of BHT, respectively. Otherwise, HS extracts I–III showed similar and very high antioxidant activity (Table 3). 50% Ethanol HS extract II has the highest antioxidant activity showing similar results with study that solvent ethanol/water 60/40 (*v*/*v*) was the most suitable to get flaxseed extract with the highest antioxidant activity [40]. Actually, the antioxidant activity of I–III is more than 15 times higher than for the roasted Tombul hazelnut skin (EC_50_ = 1.01 mg/mL) [8] and I–III extracts show almost the same efficiency to scavenge DPPH radical as antioxidant vitamin C with EC_50_ of 0.046 mg/mL (Table 3). High antioxidant activity of HS extracts might be attributed to condensed tannins content in hazelnut skin [41]. Some research groups studied different extraction methods and different roasted conditions for HS and EC_50_ values were in the range from 1.12 to 4.02 mg/mL and from 3.67 to 10.11 µg/mL, respectively [26,42].

### 3.7. Quantitative Analysis of the Identified Phenolic Compounds by HPLC-ESI-MS/MS

HPLC-ESI-MS/MS analyses of HS extracts I–III identified 10 phenolic compounds by using mixture of 20 phenolic standards. Concentrations of determined phenolic compounds in HS extracts I–III are shown in Table 4. Yield of gallic acid is the highest, compared with other three phenolic acids: protocatechuic, *p*-hydroxybenzoic and ferulic in extracts I–III (Table 4). These results are in close agreement with reported studies of Alasavar et al. for varieties of natural and roasted Turkish hazelnut skins [8]. Pelvan et al. also showed that gallic acid and protocatechuic acid were the first two dominant phenolic acids in seven commercial varieties of roasted Turkish hazelnuts [43], whereas Del Rio et al. confirmed protocatechuic acid as the predominant one in hazelnut skins [44]. The highest values of phenolic acids concentrations in HS extracts were obtained in III, that is in agreement with its highest value of TPC (Table 3 and Table 4). Six flavonoid compounds were determined in I–III, with catechin as the most abundant in all HS extracts (939–1778 µg/g), followed by epicatechin, quercetin, rutin, naringenin and resveratrol in I and with slightly different order in II and III (Table 4). HS extract III also showed the highest concentrations of all flavonoids although it has the lowest values of TFC and antioxidant activity (Table 3 and Table 4).

### 3.8. Fatty Acids Composition

The fatty acid composition of hexane and chloroform/methanol TL extracts are given in Table 5. The most abundant fatty acids were oleic acid followed by linoleic acid and saturated fatty acids such as palmitic and stearic acids indicating similar values with reported data [45,46,47]. Content of unsaturated oleic acid has slightly higher value and contents of saturated palmitic and stearic acids have slightly lower values in hexane TL extract due to the nonpolar solvent compared to chloroform/methanol TL extract. 

### 3.9. Determination of In Vitro SPF by UV Spectroscopy

**Figure 2:**
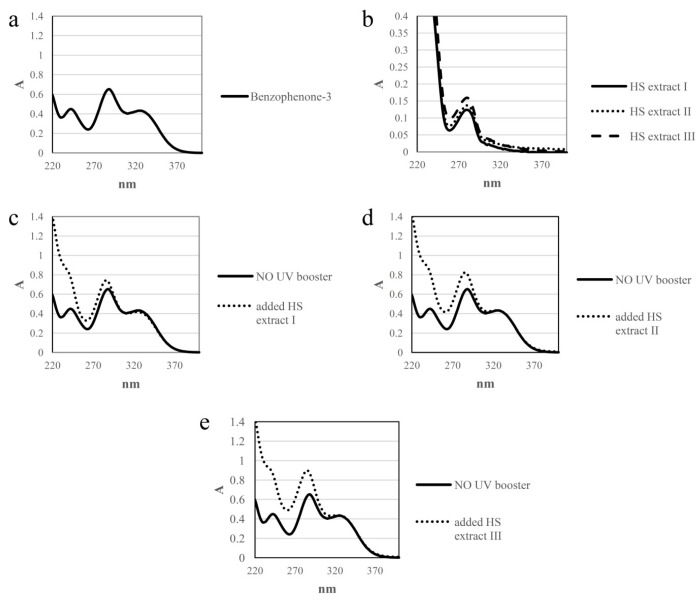
UV spectra of (**a**) 10 mg/L of benzophenone-3, (**b**) 10 mg/L of HS extracts I–III, (**c**) 10 mg/L benzophenone-3 solution boosted with 10 mg/L of HS extracts I, (**d**) 10 mg/L benzophenone-3 solution boosted with 10 mg/L of HS extracts II, (**e**) 10 mg/L benzophenon-3 solution boosted with 10 mg/L of HS extracts III.

UV-spectra of sunscreen agent benzophenone-3 and HS extracts I–III at concentration of 10 mg/L are shown in Figure 2a,b. UV filter benzophenone-3 showed significantly higher absorption value in UVB region (290–320 nm) compared with extracts I–III, that is agreement with results of Galanakis et al. obtained for benzophenone-3 and olive phenols recovered from olive mill waste water with same concentration [16,17].Compared to olive phenols that showed higher absorption values in the UVA region (340–400 nm) than UV filter benzophenone-3, our HS extracts I–III did not absorb at all between 350 and 400 nm.

Figure 2c–e illustrate UV spectra of sunscreen agentbenzophenone-3 solutions (10 mg/L) boosted with of HS extracts I–III (10 mg/L). HS extracts increased slightly the absorption of benzophenone-3 in UVB region in the following order: I <II < III. Extract III showed the highest absorption due to the highest value of TPC, as well as the highest contents of phenol and flavonoid compounds (Table 3 and Table 4). Compared to results of Galanakis et al. that obtained significant increase of UVB absorption of benzophenone-3 boosted with olive phenols (10 mg/L), the boosted effect of our HS extracts I–III is negligible [17].

Table 6 shows *in vitro* SPF values calculated from UV spectra of sunscreen agent benzophenone-3, HS extracts I–III and benzophenone-3 boosted with extracts I–III, with same concentration (10 mg/L). Indeed, all extracts increased slightly the SPF value of benzophenone (4.66–4.94) compared to 4.35 for the single solution. Extract III showed the highest SPF value, regarding the highest concentrations of phenols and flavonoids (Table 4). On the other side, Galanakis et al. observed almost twice higher SPF value of benzophenone-3 boosted with olive phenols than single solution [17]. Low SPF values of our HS extracts I–III indicated that they cannot be used as UV-boosters in cosmetics.

## 4. Conclusions

Study of various ethanol extracts of HS with of Serbian origin showed that the highest yield of phenolic compounds was obtained in 96% ethanol HS extract III, while the highest yield of flavonoids and antioxidant activity were achieved in 50% ethanol HS extract II. Roasted HS contained high content of total phenols, flavonoids, and significant antioxidant activity indicating its importance in application as functional food ingredients and nutrients. Determination of 10 phenolic compounds in the HS extracts I–III showed that gallic acid and catechin, very important antioxidants, were the most abundant phenolic acid and flavonoid, respectively. CHN% and metal contents of HS, as well as richness in unsaturated fatty acids such as oleic and linoleic acid, improve its nutritional value promoting either human health or fungal and microbial growth. Finally, the present study demonstrated that based on very high C/N, C/P and C/N/P ratios, as well as, CHN% of HS that varies in the range of CHN% for fungal biomass, roasted HS can find potential application as nutrient for fungal growth. Although HS extracts I–III have shown high antioxidant activity and high contents of phenols and flavonoids, our study of their possible application as UV booster, indicating their negligible boosted effect on sunscreen agent benzophenone-3.

## Figures and Tables

**Table 1:** Proximate composition of roasted hazelnut skins (HS) sample.

Roasted HS (%)	
Moisture	8.56 ± 0.06
Lipids (dw)	12.03 ± 0.13
Proteins (dw)	7.43 ± 0.08
Ashes (dw)	1.80 ± 0.02
Carbohydrates (dw)	70.16 ± 1.12

Results are expressed as mean ± SD (*n* = 3).

**Table 2:** Essential and nonessential minerals contents of HS sample.

Mineral	Units	Mean ± SD
Aluminium	mg/100 g	1.628 ± 0.133
Antimony	µg/100 g	23.183 ± 2.265
Arsenic	mg/100 g	ND
Barium	mg/100 g	0.379 ± 0.019
Boron	mg/100 g	2.796 ± 0.020
Cadmium	µg/100 g	7.438 ± 0.127
Calcium	mg/100 g	85.804 ± 0.655
Chromium	µg/100 g	24.739 ± 14.740
Cobalt	mg/100 g	ND
Copper	mg/100 g	0.440 ± 0.004
Iron	mg/100 g	4.191 ± 0.127
Lead	mg/100 g	ND
Lithium	mg/100 g	9.783 ± 0.285
Magnesium	mg/100 g	113.780 ± 1.252
Manganese	mg/100 g	1.457 ± 0.028
Mercury	mg/100 g	ND
Nickel	µg/100 g	72.937 ± 5.395
Phosphorous	mg/100 g	52.557 ± 0.304
Potassium	mg/100 g	15.920 ± 0.307
Selenium	µg/100 g	23.865 ± 4.316
Sodium	mg/100 g	60.462 ± 1.325
Strontium	mg/100 g	1.195 ± 0.004
Zinc	mg/100 g	1.107 ± 0.012

Values represent mean ± SD (*n* = 3). ND: No Detected.

**Table 3:** Total phenols contents (TPC), total flavonoids contents (TFC), antiradical activity DPPH) (EC_50_, mg/mL) and equivalents of vitamin C (EC_50_ = 0.046 mg/mL) of HS extracts I–III.

Samples	TPC (mg GAE/g)	TFC (mg CE/g)	DPPH (EC_50_, mg/mL)	Equivalents of Vitamin C
I EtOH/H_2_O 10%	430.7 ± 21.0 ^a^	538.2 ± 26.4 ^a^	0.062 ± 0.006 ^a^	0.742
II EtOH/H_2_O 50%	588.0 ± 5.9 ^b^	549.9 ± 18.0 ^a^	0.052 ± 0.002 ^b^	0.885
III EtOH/H_2_O 96%	706.0 ± 9.7 ^c^	477.7 ± 28.6 ^b^	0.066 ± 0.001 ^a^	0.697

Results are expressed as mean ± SD (*n* = 3). Means ± SD followed by the same letter, within a column are not significant different (*p* > 0.05).

**Table 4:** Concentrations of identified phenolic compounds in HS extracts I–III by HPLC-ESI-MS/MS.

	Concentration in Extracts μg/g		
	I	II	III
Galic acid	998 ± 228 ^a^	774 ± 228 ^a^	1168 ± 381 ^a^
Protocatechuic acid	841 ± 120 ^a^	664 ± 188 ^a^	937 ± 194 ^a^
*p*-Hydroxybenzoic acid	53.0 ± 14.4 ^a^	38.4 ± 13.5 ^a^	57.0 ± 16.9 ^a^
Ferulic acid	15.4 ± 4.4 ^a^	14.4 ± 3.9 ^a^	16.5 ± 3.3 ^a^
Catechin	939 ± 123 ^a^	1126 ± 160 ^a^	1778 ± 235 ^b^
Epicatechin	181 ± 23 ^a^	208 ± 28 ^a^	311 ± 6 ^b^
Quercetin	106 ± 33 ^a^	222 ± 31 ^a^	336 ± 69 ^c^
Rutin	29.4 ± 11.8 ^a^	32.1 ± 7.7 ^a^	31.2 ± 9.1 ^a^
Resveratrol	5.6 ± 6.5 ^a^	14.7 ± 1.1 ^b^	22.1 ± 1.9 ^b^
Naringenin	20.4 ± 6.8 ^a^	28.8 ± 4.4 ^a^	39.6 ± 10.4 ^a^

Data are expressed as the mean ± SD (*n* = 3). Means ± SD followed by the same letter, within the same row, are not significantlydifferent (*p >* 0.05).

**Table 5:** Fatty acid composition (g/100g of total fatty acids) of roasted HS in Serbia.

Fatty Acid	% (g/100g) ± SD	
	Hexane Extract	CHCl_3_/MeOH Extract
C16:0	6.05 ± 0.08 ^a^	6.33 ± 0.38 ^a^
C18:0	2.09 ± 0.02 ^a^	2.24 ± 0.20 ^a^
C20:0	0.16 ± 0.05 ^a^	0.14 ± 0.01 ^a^
C16-1n-7	0.15 ± 0.01 ^a^	0.16 ± 0.01 ^a^
C18-1n-9	74.81 ± 0.35 ^a^	73.72 ± 0.65 ^a^
C20-1n-9	0.18 ± 0.01 ^a^	0.20 ± 0.01 ^a^
C18-2n-6	16.13 ± 0.10 ^a^	16.33 ± 0.15 ^a^
C18-3n-3	0.17 ± 0.01 ^a^	0.21 ± 0.02 ^b^

Results are expressed as mean ± SD (*n* = 3). Means ± SD followed by the same letter, within the same row, are not significant different (*p* *>* 0.05).

**Table 6:** In vitrosun protection factor (SPF) of combination of sunscreen agent benzophenone-3 and HS extracts I–III as UV-boosters.

Concentration of Sunscreen Agent	UV-Protection Booster (10 mg/L)	In VitroSPF
10	-	4.35 ± 0.16 ^a^
-	I	0.24 ± 0.07 ^b^
-	II	0.28 ± 0.04 ^b^
-	III	0.31 ± 0.08 ^b^
10	I	4.66 ± 0.03 ^c^
10	II	4.85 ± 0.10 ^c^
10	III	4.94 ± 0.20 ^c^

Values represent mean ± SD (*n* = 3). ^a–c^ Values having the same superscripted letter within a column are not significantly different (*p* > 0.05).
